# Mild hyperphenylalaninemia (hpa) presenting as orthostatic tremor: a case report

**DOI:** 10.1186/s12883-022-02946-1

**Published:** 2022-11-04

**Authors:** Hua Li, Hua Yang, Min Li, Li Liang, Haojing Zhu, Anan Chen, Hairong Qian

**Affiliations:** 1 Senior Department of Neurology, the First Medical Center of PLA General Hospital, NO.28 Fuxing Road, HaiDian District, Beijing, 100853 China; 2 Navy Clinical College, the Fifth School of Clinical Medicine, Anhui Medical University, Hefei, 230032 Anhui China; 3 The Second School of Clinical Medicine, Southern Medical University, Guangzhou, 510515 Guangdong China

**Keywords:** Hyperphenylalaninemia, Orthostatic tremor, Case report, Electromyography, Compound heterozygous mutation

## Abstract

**Background:**

Orthostatic tremor (OT) is a type of postural tremor of the lower extremities that has not been described in either phenylketonuria (PKU) or hyperphenylalaninemia (HPA). Because little is known about the clinical features and therapeutic responses of OT in mild HPA, we describe a mild HPA patient who presented with OT as an initial symptom.

**Case presentation:**

A 22-year-old male was admitted for bilateral leg tremor while standing, with symptom onset eight months prior. One month before admission, the tremor disappeared in the left leg but persisted in the right leg. Electromyography recorded from the right gastrocnemius revealed a 6–8 Hz tremor, which appeared when the patient was standing and disappeared when he was resting or walking. Blood screening showed a phenylalanine/tyrosine ratio of 2.06 and a phenylalanine level of 140 μmol/L. Urine metabolic screening was negative. Whole-exome sequencing confirmed the presence of a compound heterozygous mutation, c.158G > A and c.728G > A, in phenylalanine hydroxylase (PAH) gene. After three months of levodopa/benserazide tablets (250 mg, tid) and a low-phenylalanine diet treatment, the tremor disappeared.

**Conclusions:**

Young-onset mild HPA is a relatively rare autosomal recessive metabolic disease, and slow OT is a rare clinical feature. Metabolic screening and genetic testing are the keys to early diagnosis and treatment. For adolescents and young adults, appropriate medication and long-term dietary therapy remain important treatments. This case expanded the disease spectrum of slow OT.

**Supplementary Information:**

The online version contains supplementary material available at 10.1186/s12883-022-02946-1.

## Background

Mild hyperphenylalaninemia (HPA) is a phenotype of phenylketonuria (PKU), which can be divided into three types, namely, typical PKU, mild PKU, and mild HPA, based on the blood concentration of phenylalanine before treatment [1]. Tremor is found in 5% to 28% of PKU patients [2]. Most of these patients present with high-frequency postural and kinetic tremor of the distal upper extremities, with a rare occurrence of lower extremity tremor [3]. Orthostatic tremor (OT) is a type of postural tremor of the lower extremities that has not been described in either PKU or HPA. Because little is known about the clinical features and therapeutic responses of OT in mild HPA, we describe a mild HPA patient who presented with OT as an initial symptom.

## Case presentation

A 22-year-old man presented with leg tremor in standing position for the previous 8 months. This tremor was initially bilateral, but 7 months after development, it subsided in the left leg and persisted only in the right leg. No tremor was evident when the patient was walking, reclining, or in a non-weight-bearing position. He denied any falls and had no unsteadiness. There were no other complaints, and the patient had no significant medical history or family history of tremor.

**Fig. 1:**
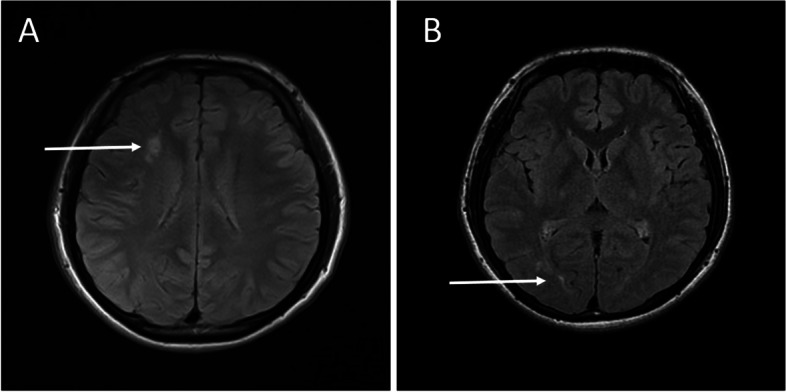
T2 brain MRI revealed mild, asymmetrical, non-enhancing white matter lesions, mainly in the right frontal **(A**) and posterior (**B**) periventricular regions

**Fig. 2:**
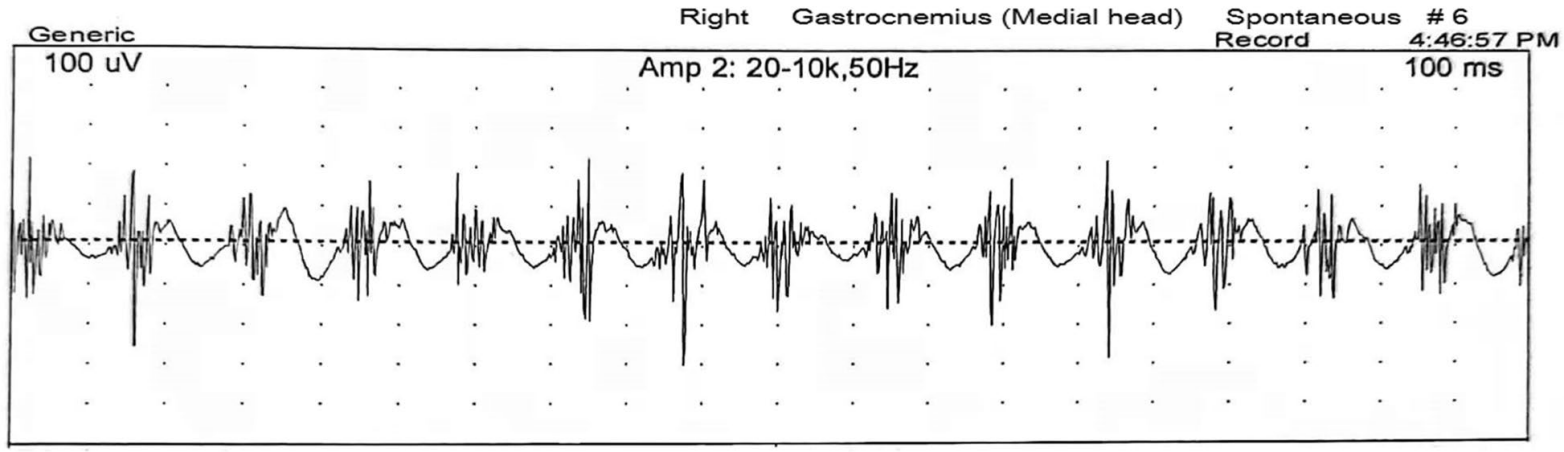
EMG recording from the lower limbs while the patient was standing. Recordings from the right gastrocnemius muscle revealed a 6–8 Hz tremor

On examination, he showed incessant quivering in his right leg while standing, but the tremor disappeared when he was asked to walk, sit, lie down, or lean against the wall for support (Video 1). There was no resting or postural tremor of upper limbs. Bilateral tendon reflexes were diminished, and there was no plantar response. Auscultation over the right popliteal fossa while the patient was standing revealed the “helicopter sign”, a sound similar to a helicopter, indicating OT. All other components of neurological examination were normal. Laboratory tests, including routine blood tests, blood biochemistry, vitamin B12, thyroid hormone, rheumatoid factor, antinuclear autoantibody, anti-cardiolipin antibody, and anti-neutrophil cytoplasmic antibody tests, were normal. Furthermore, the tests of the blood and cerebrospinal fluid samples, including tests for anti-paraneoplastic antibodies, autoimmune antibodies and anti-ganglioside antibodies, were all normal. Brain MRI revealed mild, asymmetrical, non-enhancing white matter lesions, mainly in the right frontal and posterior periventricular regions, on T2-weighted imaging (Fig. 1). No abnormal signal was found in spinal cord on MRI. Chest CT was normal except for a residual thymus gland. Fluorodeoxyglucose (FDG)-PET was performed, and no abnormal metabolism was identified. Electroencephalography (EEG) was normal. Electromyography (EMG) recorded from the right gastrocnemius revealed a 6–8 Hz tremor, which was present when the patient was standing and disappeared when he was resting or walking (Fig. 2), and no abnormalities were found in the left leg. These features were consistent with slow OT.

Considering the clinical presentation and imaging findings, blood and urine metabolic screening was performed. These tests showed a phenylalanine/tyrosine ratio of 2.06 and a blood phenylalanine level of 140 μmol/L, while there was no phenylalanine in the urine. Whole-exome sequencing with next-generation sequencing (NGS) technology confirmed the presence of a compound heterozygous mutation in c.158G > A and c.728G > A of the phenylalanine hydroxylase (PAH) gene. Pedigree verification showed that the compound heterozygous variants were inherited from the parents as follows: c.158G > A from his mother and c.728G > A from his father. The patient’s final diagnosis was OT secondary to mild HPA.

Our patient had not received any relevant interventions prior to admission. Based on the above diagnosis, we tried clonazepam (1 mg, qd), carbamazepine (100 mg, qd), and trihexyphenidyl (2 mg, tid) in sequence, but there was no clinical response to these treatments. After discharge, the patient was treated with levodopa/benserazide tablets (250 mg, tid) and a low-phenylalanine diet. One month later, the tremor was markedly alleviated. Three months later, the tremor disappeared (Table 1).

**Table 1:** Treatment and effects

Timeline	Treatment	Assessment
D10 Admission	Clonazepam (1 mg, qd) for 7 days	Absent
D17 Admission	Carbamazepine (100 mg, qd) for 7 days	Absent
D24 Admission	Trihexyphenidyl (2 mg, tid) for 7 days	Absent
D31 Admission	Levodopa/benserazide tablet (250 mg, tid), low-phenylalanine diet for 1 day	Absent
Follow-up visit 1 month after discharge	Levodopa/benserazide tablet (250 mg, tid), low-phenylalanine diet for 30 days	The tremor alleviated obviously
Follow-up visit 3 months after discharge	Levodopa/benserazide tablet (250 mg, tid), low-phenylalanine diet for 90 days	The tremor disappeared

## Discussion and conclusion

PKU was first reported in 1934 and is caused by mutation of PAH gene, which results in the accumulation of phenylalanine to neurotoxic levels. Patients with blood phenylalanine concentrations of 120–600 μmol/L before treatment are classified as having mild HPA [1]. PAH function and activity depend on the mutation site, with different sites resulting in different severities of clinical phenotypes. Clinical symptoms of PAH c.728G > A mutation are relatively mild, often emerging in adulthood, and are never reported as OT. Tremor in the lower limbs or trunk in a standing position is called OT [4]. Slow OT has a tremor frequency below 13 Hz and is frequently associated with some other neurological signs. Differential diagnosis of slow OT include Parkinson’s disease, encephalitis, hyperthyroidism, orthostatic myoclonus syndrome, etc. In this case, neither the clinical characteristics nor the laboratory results supported the presence of these diseases. According to the consensus statement on tremor classification of the Tremor Working Group of the International Parkinson's and Dyskinesia Association [4], the patient was ultimately diagnosed with slow OT secondary to mild HPA.

Slow OT still has an unclear pathogenetic origin; theories include a central oscillatory network, an altered cerebello–thalamo–cortical network, neurodegeneration, and a dopaminergic deficit [5–7]. Although there was neither cerebellar volume change nor abnormal metabolism on FDG-PET in our case, there is an accumulating body of evidence suggesting a key role of the cerebellum and related areas in its pathophysiology. For example, Kristina A et al. reported that individuals with early-treated PKU had reduced cerebellar grey matter volume, which was impacted by PKU, and M. Seki found that N-isopropyl-P-[^123^I]iodoamphetamine (^123^I-IMP) single-photon emission computed tomography (SPECT) showed diffuse hypoperfusion in the cerebellum in PKU patients [8, 9]. On the other hand, phenylalanine is transported by L-amino acid transporter 1 (LAT1). Precursors of tyrosine, dopamine and norepinephrine are also transported by LAT1. A high blood concentration of phenylalanine can competitively inhibit the function of this transporter, thus affecting the synthesis of a variety of intracranial neurotransmitters, such as dopamine, leading to neural dysfunction [1]. This would be a possible mechanism for HPA-induced damage to the brain. In PKU, the ability to convert phenylalanine to tyrosine is reduced, leading to an increased concentration of phenylalanine and a decreased concentration of tyrosine. As the phenylalanine: tyrosine ratio increases, dopamine levels may decrease. Based on the above reasons, we tried levodopa/benserazide (250 mg, tid) and a low-phenylalanine diet for treatment and achieved good results, which verified that dopamine deficiency was part of the mechanism.

Current American and European guidelines covering all aspects of PKU recommend starting treatment at blood phenylalanine concentrations above 360 μmol/L [10, 11]. Current US guidelines are based on the idea that the closer the phenylalanine concentration is to the physiological level, the better the outcome will be [12]. Dietary phenylalanine restriction is the main therapy. In this case, the clinical manifestations of OT appeared when the concentration of phenylalanine was only 140 μmol/L. After one month of a low-phenylalanine diet and levodopa/benserazide tablet treatment, the tremor symptoms improved significantly. Therefore, it is beneficial for patients to start treatment when they have clinical symptoms, even if their blood phenylalanine concentration is below 360 μmol/L.

On follow-up, no measurement of phenylalanine concentration was obtained after resolution of the tremor, which makes it difficult to further verify the pathogenesis; this might be one limitation of the present case report. In this case, the patient's symptoms were not alleviated immediately after levodopa/benserazide administration, which might be related to the low starting dose of dopamine and the long-term neurotoxicity of HPA. Relief of tremor symptoms might not occur until the tyrosine level falls to a sufficiently low level. Decreased blood tyrosine levels might have played a key role in the disappearance of this patient's symptoms after a relatively long period of treatment with a low-phenylalanine diet. This is consistent with our hypothesis regarding the pathogenetic mechanism. Whether the levodopa/benserazide treatment or the low-phenylalanine diet treatment played a more important role in the disappearance of slow OT symptoms in this patient needs to be further investigated.

Mild HPA with young onset is a relatively rare autosomal recessive metabolic disease, with slow OT as a rare clinical feature. Metabolic screening and genetic testing are the keys to early diagnosis and treatment. For adolescents and young adults, appropriate medication and long-term dietary therapy remain important treatments.

## Supplementary Information

**Additional file 1:** **Video 1.** This video demonstrates key clinical features of the tremor. Our patient showed incessant quivering in his right lower limb while standing, but the movement disappeared when he was asked to walk, sit, lie down, or lean against the wall for support.

## Data Availability

The data used within this article will be made available from the corresponding authors on reasonable request.

## Abbreviations

HPA: Mild hyperphenylalaninemia
OT: Orthostatic tremor
PKU: Phenylketonuria
PAH: Phenylalanine hydroxylase
CSF: Cerebrospinal fluid
MRI: Magnetic resonance imaging
T2WI: Magnetic resonance T2 weighted image
CT: Computed tomography
EMG: Electromyography
EEG: Electroencephalogram
NGS: Next generation sequencing
LAT1: L-amino acid transporter 1

## References

[1] Blau N, van Spronsen FJ, Levy HL (2010). Phenylketonuria. The Lancet.

[2] Perez-Duenas B, Valls-Sole J, Fernandez-Alvarez E, Conill J, Vilaseca MA, Artuch R (2005). Characterization of tremor in phenylketonuric patients. J Neurol.

[3] Jaulent P, Charriere S, Feillet F, Douillard C, Fouilhoux A, Thobois S (2020). Neurological manifestations in adults with phenylketonuria: new cases and review of the literature. J Neurol.

[4] Bhatia KP, Bain P, Bajaj N, Elble RJ, Hallett M, Louis ED (2018). Consensus Statement on the classification of tremors. from the task force on tremor of the International Parkinson and Movement Disorder Society. Mov Disord.

[5] Benito-Leon J, Rodriguez J, Orti-Pareja M, Ayuso-Peralta L, Jimenez-Jimenez FJ, Molina JA (1997). Symptomatic orthostatic tremor in pontine lesions. Neurology.

[6] Benito-Leon J, Domingo-Santos A (2016). Orthostatic Tremor: An Update on a Rare Entity. Tremor Other Hyperkinet Mov (N Y).

[7] Benito-Leon J, Romero JP, Louis ED, Sanchez-Ferro A, Matarazzo M, Molina-Arjona JA (2019). Diffusion tensor imaging in orthostatic tremor: a tract-based spatial statistics study. Ann Clin Transl Neurol.

[8] Aldridge K, Cole KK, Moffitt Gunn AJ, Peck D, White DA, Christ SE (2020). The effects of early-treated phenylketonuria on volumetric measures of the cerebellum. Mol Genet Metab Rep.

[9] Seki M, Takizawa T, Suzuki S, Shimizu T, Shibata H, Ishii T (2015). Adult phenylketonuria presenting with subacute severe neurologic symptoms. J Clin Neurosci.

[10] van Spronsen FJ, van Wegberg AMJ, Ahring K, Bélanger-Quintana A, Blau N, Bosch AM (2017). Key European guidelines for the diagnosis and management of patients with phenylketonuria. Lancet Diabetes Endocrinol.

[11] Vockley J, Andersson HC, Antshel KM, Braverman NE, Burton BK, Frazier DM (2014). Phenylalanine hydroxylase deficiency: diagnosis and management guideline. Genet Med.

[12] van Spronsen FJ, Blau N, Harding C, Burlina A, Longo N, Bosch AM (2021). Phenylketonuria. Nat Rev Dis Primers.

